# Effectiveness of Live Health Professional–Led Group eHealth Interventions for Adult Mental Health: Systematic Review of Randomized Controlled Trials

**DOI:** 10.2196/27939

**Published:** 2022-01-11

**Authors:** Cheryl L Currie, Richard Larouche, M Lauren Voss, Maegan Trottier, Rae Spiwak, Erin Higa, David R Scott, Treena Tallow

**Affiliations:** 1 Faculty of Health Sciences University of Lethbridge Lethbridge, AB Canada; 2 Department of Surgery Max Rady College of Medicine University of Manitoba Winnipeg, MB Canada; 3 Library Services University of Lethbridge Lethbridge, AB Canada; 4 Alberta Health Services Lethbridge, AB Canada

**Keywords:** systematic review, telemedicine, eHealth, mHealth, e-therapy, mobile interventions, internet, adult, mental health, anxiety, depression, substance use, bereavement, physical activity, CBT, psychotherapy, group, synchronous, videoconference, teleconference

## Abstract

**Background:**

The COVID-19 pandemic has had adverse impacts on mental health and substance use worldwide. Systematic reviews suggest eHealth interventions can be effective at addressing these problems. However, strong positive eHealth outcomes are often tied to the intensity of web-based therapist guidance, which has time and cost implications that can make the population scale-up of more effective interventions difficult. A way to offset cost while maintaining the intensity of therapist guidance is to offer eHealth programs to groups rather than more standard one-on-one formats.

**Objective:**

This systematic review aims to assess experimental evidence for the effectiveness of live health professional–led group eHealth interventions on mental health, substance use, or bereavement among community-dwelling adults. Within the articles selected for our primary aim, we also seek to examine the impact of interventions that encourage physical activity compared with those that do not.

**Methods:**

Overall, 4 databases (MEDLINE, CINAHL, PsycINFO, and the Cochrane Library) were searched in July 2020. Eligible studies were randomized controlled trials (RCTs) of eHealth interventions led by health professionals and delivered entirely to adult groups by videoconference, teleconference, or webchat. Eligible studies reported mental health, substance use, or bereavement as primary outcomes. The results were examined by outcome, eHealth platform, and intervention length. Postintervention data were used to calculate effect size by study. The findings were summarized using the Synthesis Without Meta-Analysis guidelines. Risk of bias was assessed using the Cochrane Collaboration Tool.

**Results:**

Of the 4099 identified studies, 21 (0.51%) RCTs representing 20 interventions met the inclusion criteria. These studies examined mental health outcomes among 2438 participants (sample size range: 47-361 participants per study) across 7 countries. When effect sizes were pooled, live health professional–led group eHealth interventions had a medium effect on reducing anxiety compared with inactive (Cohen *d*=0.57) or active control (Cohen *d*=0.48), a medium to small effect on reducing depression compared with inactive (Cohen *d*=0.61) or active control (Cohen *d*=0.21), and mixed effects on mental distress and coping. Interventions led by videoconference, and those that provided 8-12 hours of live health professional–led group contact had more robust effects on adult mental health. Risk of bias was high in 91% (19/21) of the studies. Heterogeneity across interventions was significant, resulting in low to very low quality of evidence. No eligible RCT was found that examined substance use, bereavement, or physical activity.

**Conclusions:**

Live eHealth group interventions led by health professionals can foster moderate improvements in anxiety and moderate to small improvements in depression among community-based adults, particularly those delivered by videoconference and those providing 8-12 hours of synchronous engagement.

**Trial Registration:**

PROSPERO CRD42020187551; https://www.crd.york.ac.uk/prospero/display_record.php?RecordID=187551

**International Registered Report Identifier (IRRID):**

RR2-10.1186/s13643-020-01479-3

## Introduction

### Background

The COVID-19 pandemic has resulted in many forms of loss. These privations have had adverse impacts on adult mental health and substance use worldwide [1-3]. Scalable interventions are needed to address the immediate-, mid-, and long-term psychological consequences of the pandemic across countries [4]. Mental health interventions delivered by video, telephone, and mobile apps, termed eHealth interventions, are critical and effective tools to address this need [5-12].

When interventions are delivered using eHealth platforms, patient engagement in the intervention and clinical outcomes are often tied to the intensity of the interaction between clinicians and their clients [8]. For example, a systematic review found that one-on-one therapist-supported internet cognitive behavioral therapy (CBT) was more effective than attention, information, or web-based discussion group controls at reducing adult anxiety [13]. Similarly, a 2021 randomized controlled trial (RCT) found that live, therapist-guided web-based therapy was superior to an unguided form of the program delivered by email at improving pandemic-induced anxiety and depression [14]. A review of best practices to improve engagement and adherence in eHealth interventions specifically recommends the inclusion of human, or a sense of human, contact to build a therapeutic alliance with clients and help them feel more accountable to engage in the interventions [15].

A pair of 2019 systematic reviews concluded that health professional–led videoconference interventions are effective at improving adult anxiety and depression when delivered live and one-on-one between a therapist and a client [11,12]. Yet, therapist engagement in eHealth interventions has time and cost implications that can make the population scale-up and accessibility of more effective programs difficult [16]. A way to offset time and cost while maintaining the intensity of therapist guidance is to offer live therapist-led eHealth interventions to *groups* rather than one-on-one. If effective, group-based eHealth interventions could expand public access to mental health professionals during and after the pandemic.

In Canada and other countries, there is also a need for eHealth interventions that are accessible and culturally safe for Indigenous people and remote communities in addition to the general population [17]. Group-delivered interventions may be more in keeping with the community-centered focus of many Indigenous cultures and thus may be more culturally appropriate for Indigenous clients and communities [18,19]. Group-delivered mental health and substance use interventions have been shown to have cultural utility for Indigenous people, while also providing benefits for non-Indigenous people seeking help for these problems [20,21]. This is not surprising, given that humans are social creatures. Our lives are shaped by our experiences in groups, making the propensity to congregate a powerful therapeutic tool [22]. Group interventions have been shown to promote client engagement in treatment through rewarding and therapeutic forces such as affiliation, support, empathy, and identification [22]. Group interventions can also enable those struggling with mental health and addiction to witness and strive for the healing they see in others, as well as reduce the sense of isolation that mental health and addiction problems can create [23-25].

The need for a systematic review to understand whether group health professional–led interventions could be delivered effectively using eHealth platforms became apparent to our research team in 2020. The year before, we had launched an RCT to assess the mental health impacts of health professional–led interventions delivered to groups in person [26]. In March 2020, we stopped the RCT abruptly because of rapidly spreading COVID-19 in our region and public health restrictions on indoor gatherings. Our in-person mental health interventions had been carefully designed over many months using the principles of patient-oriented research, defined as a process that engages patients and providers, focuses on patient-relevant priorities, and seeks to improve health care practices to improve patient outcomes [27]. When pandemic restrictions required that our interventions move to the web, our Indigenous, patient, and clinical partners recommended that group delivery be maintained. Thus, we sought a systematic review in the literature to guide our efforts. We found 2 systematic reviews that examined this evidence specifically for videoconference-delivered interventions [28,29]. Both concluded that they were feasible for, and well accepted by, adults. Both reviews also observed a trend toward mental health improvement. However, the teams were not confident in their observations, given that they did not specifically search for mental health outcomes. As well, most studies included in these reviews were observational, and interventions delivered by teleconference and live chat platforms were excluded. Thus, we conducted a systematic review of RCTs specifically focused on mental health outcomes for live, health professional–led group interventions delivered by videoconference, teleconference, or live chat platforms both to inform our own work and the work of others.

### Review Aims

The primary aim of this systematic review is to assess experimental evidence for the effectiveness of live health professional–led group eHealth interventions on mental health, substance use, or bereavement among community-dwelling adults. Bereavement and loss were included as outcomes for this review in light of the increased morbidity and mortality that many populations have experienced during the COVID-19 pandemic. A 2020 systematic review of 7 RCTs concluded that web-based one-on-one bereavement interventions are promising [30]. However, the role that group eHealth interventions could play in addressing bereavement outcomes is unknown. Within articles selected for our primary aim, we also sought to examine the impact of eHealth interventions that encouraged physical activity compared with those that did not, given that physical activity has been shown to improve adult mental health [31-33].

## Methods

### Protocol and Registration

This review was conducted according to the PRISMA (Preferred Reporting Items for Systematic Reviews and Meta-Analyses) statement [16,34]. The review protocol was registered with PROSPERO (CRD42020187551) and published as a protocol [35]. Ethics approval and participant consent were not required, given that the review was based on data from previously published studies.

### Eligibility Criteria

#### Eligible Study Designs

This review was limited to RCTs. Nonrandomized and observational studies were excluded. The included studies compared eligible interventions with inactive control interventions (placebo, no treatment, usual or standard care, or a waiting list control) or active control interventions that differed from the treatment intervention (eg, a different variant of the same intervention or a different kind of therapy) in keeping with recommendations from the Cochrane Handbook [36]. Active control interventions included those that were unguided, individual, or delivered in person.

#### Eligible Interventions

To be eligible, interventions had to be made up of ≥3 sessions delivered entirely live to groups on the web using a video or chat platform or by teleconference. Interventions had to be led by a health facilitator with professional training related to the intervention. This was defined as a certificate or degree in medicine, nursing, allied health, counseling, psychology, social work, or alternative health therapies. Interventions were excluded if they were not delivered in a live, synchronous group format and were not delivered entirely on the web or by telephone. Peer-led groups and web-based groups led by individuals without a recognized certificate or degree related to the intervention were also excluded.

#### Eligible Participants

Studies that examined community-dwelling adults aged ≥18 years with self-reported or physician-diagnosed mental health, substance use, or bereavement concerns were included. Studies that examined patients in palliative care or adults living in institutionalized settings (eg, care homes, hospitals, and prisons) were excluded.

#### Eligible Outcomes

The primary outcomes were changes in (1) acute or chronic mental health conditions or concerns, (2) acute or chronic substance use conditions or concerns, or (3) bereavement. In our protocol we had proposed to examine physical health and behavioral outcomes beyond substance use [35]. However, upon reflection we recognized that our search strategy (Multimedia Appendix 1) was not designed to systematically search for these outcomes and we removed them from our review aims. To increase the utility of this review, we have summarized findings from eligible studies that reported physical or behavioral health outcomes other than substance use, with an added caution that these summaries are not based on a comprehensive search of the literature for these outcomes.

### Information Sources and Search

In all, 4 electronic databases were searched to identify relevant studies published in English or French from January 2005 to June 2020 (MEDLINE, PsycINFO, CINAHL, and the Cochrane Central Register of Controlled Trials). Reference lists of the selected articles were also searched. The search strategy was developed by a health librarian (DRS) and performed in July 2020 using a combination of key words relevant for each database.

### Study Selection and Data Extraction

The results were imported and deduplicated in Covidence (Veritas Health Innovation Ltd) [36]. Titles and abstracts were independently screened in duplicate by 4 reviewers (MLV, EH, MT, and Sydney Murdoch). Full-text screening was conducted by the same reviewers for all articles that met the eligibility criteria or had unclear eligibility. Disagreements were resolved through consensus between 2 reviewers. If a decision could not be reached, consensus was achieved by discussing with an investigator (CLC or RL). Where information in the article was unclear, the corresponding author was contacted for clarification before inclusion. If the corresponding author could not be reached, articles with unclear inclusion criteria were excluded.

Data extraction was carried out independently, in duplicate, by 4 reviewers using Covidence (MLV, EH, MT, and Sydney Murdoch). The extracted data included descriptions of the study sample, intervention details, analytic method, and relationships between the interventions and outcomes of interest. Outcomes reported as means, SDs, and effect estimates were also extracted. Where data were insufficient or not available in the published paper or not obtainable by contacting the authors, studies were excluded from the review. Of note, several RCTs examined the same intervention, reported >1 outcome, or reported >1 measure per outcome, all of which are included in this review.

### Synthesis of Results and Assessment of Heterogeneity

Given the expected heterogeneity across studies, it was determined that it would not be appropriate to conduct a meta-analysis. Instead, a narrative analysis of the studies following the Synthesis Without Meta-Analysis reporting guidelines was performed [37]. We explored heterogeneity of the intervention effects by comparing the effect sizes of studies grouped by (1) mental health outcome, (2) intervention delivery platform (videoconference, teleconference, and synchronous chat), and (3) intervention intensity (<8 contact hours, 8-12 contact hours, and >12 contact hours).

In the protocol, we had planned to assess intervention effects by sex and gender [35]. We were unable to do so because of a lack of studies that reported outcomes by these variables. In the protocol, we had also planned to compare the effects of eHealth interventions delivered to groups with those of eHealth interventions delivered to active or inactive control, with results combined across the 2 control conditions. Upon reviewing the studies selected for the review, we found differential intervention effects for active and inactive controls and have presented them separately in the results.

We found a small but significant subset of studies focused on nonprofessional caregiver mental health. Although we did not specifically search for RCTs related to caregiver mental health, these studies did meet our search criteria. Thus, our main findings summarize results for all participants, including those who are caregivers (eg, parents of children with cancer). To increase the utility of our findings, we have also provided a summary of intervention effects specifically for nonprofessional caregivers.

### Quality Assessment

We evaluated the quality of evidence for each outcome using the Grading of Recommendations Assessment, Development and Evaluation approach [38]. Outcomes were assessed using the following categories: (1) *high certainty*: we are very confident that the true effect lies close to the effect estimate; (2) *moderate certainty*: we are moderately confident that the true effect is close to the effect estimate, but there is a possibility that it is substantially different; (3) *low certainty*: we have limited confidence in the effect estimate; the true effect may be substantially different from the effect estimate; and (4) v*ery low certainty*: we have very little confidence in the effect estimate; the true effect is likely to be substantially different from the effect estimate.

Two reviewers (MLV and EH) independently rated the quality of evidence. Given that all studies were RCTs, each rating began as *high quality*. We downgraded quality by 1 level for serious concerns and by 2 levels for very serious concerns about risk of bias, inconsistency, indirectness, imprecision, and publication bias. We used the GRADEpro Guideline Development Tool (McMaster University and Evidence Prime) to generate summary of findings tables for intervention outcomes compared with active and inactive controls [39].

### Statistical Analysis

Given that no RCTs selected for this review had a sample size <20 and most had sample sizes >50, we calculated effect sizes using *Cohen d* to allow for a comparison of effects (ie, rather than *Hedges g*, which is typically used to address inflation when sample sizes are <20) [40]. Effect sizes were calculated by subtracting the mean posttest score for the treatment group from the mean posttest score for the control group and dividing the result by the pooled SD of the 2 groups [41]. Effect sizes were computed between the groups within 1 month after the intervention period, given that this time point was most consistently reported across the included studies. Effect sizes were categorized as *trivial* (Cohen *d*<0.20), *small* (Cohen *d*=0.20-0.49), *medium* (Cohen *d*=0.50-0.79), and *large* (Cohen *d*≥0.80) following the guidelines provided by Cohen [41]. Wilcoxon signed-rank tests in R were used to calculate 95% CIs for pooled effect sizes (with the exception of effect sizes corresponding to single studies, for which 95% CIs could not be produced) [42]. As recommended when a meta-analysis of effect estimates is not conducted, visual displays were created to summarize effect sizes by outcome across studies [43]. Specific effect size calculations by study are provided in Multimedia Appendix 2 [44-64].

### Risk of Bias

Risk of bias was assessed for all studies using the Cochrane Risk-of-Bias Tool [65]. Of the 21 articles, the first 5 (24%) were assessed by 4 reviewers (MLV, EH, MT, and Sydney Murdoch) and the coinvestigators (CLC, RL, and RS) and compared for consistency. Next, 4 reviewers (MLV, EH, MT, and Sydney Murdoch) independently reviewed articles for risk of bias. Judgments (high, some concerns, and low) were made based on all risk-of-bias domains.

## Results

### Study Selection

**Figure 1:**
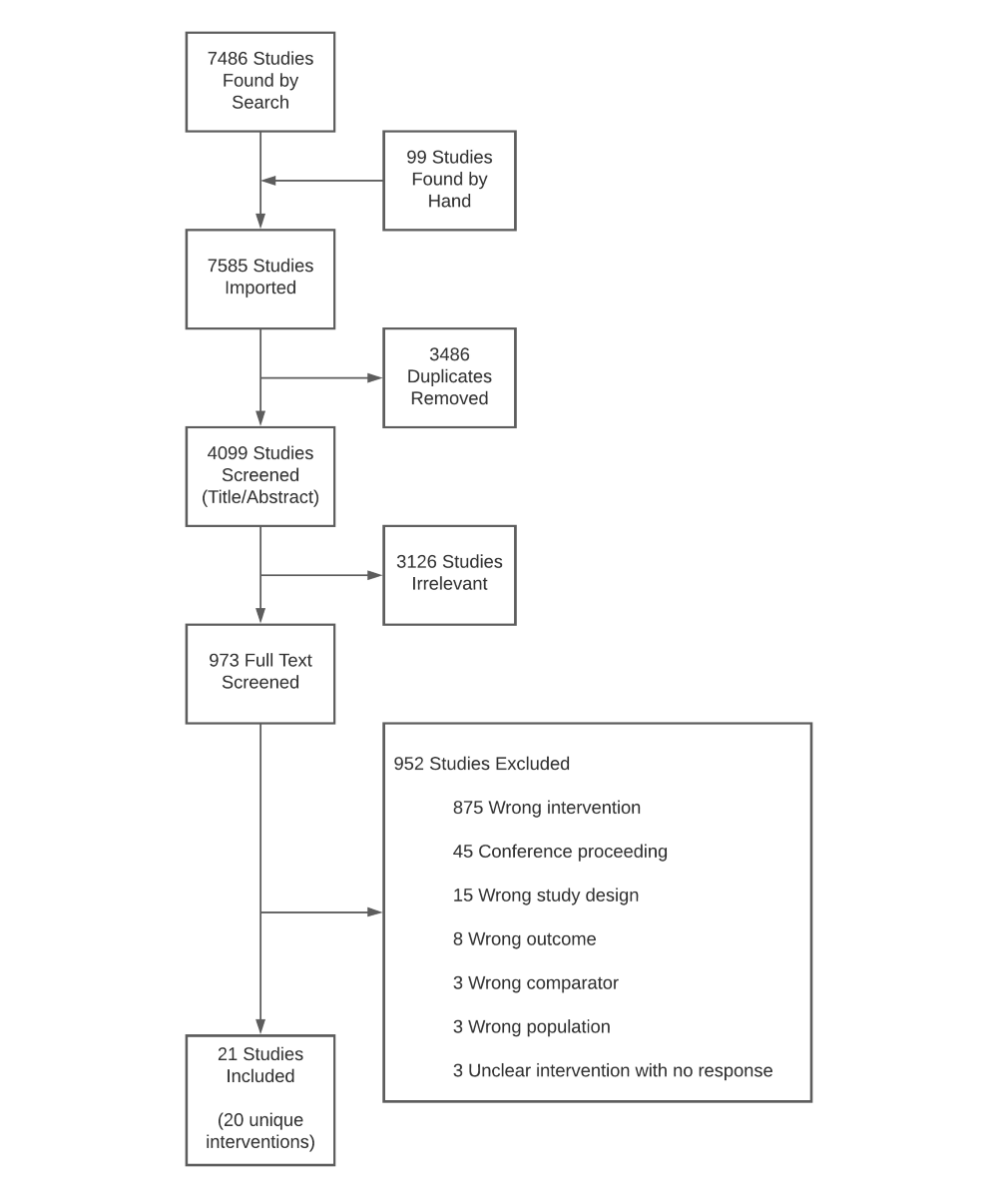
PRISMA (Preferred Reporting Items for Systematic Reviews and Meta-Analyses) flowchart.

Overall, 21 studies representing 20 interventions were included in this review. As shown in Figure 1, the search yielded 7486 articles, with an additional 99 articles included in the initial screening phase through hand searching the reference lists of identified studies. From the 7585 articles, 3486 (45.96%) duplicates were removed, leaving 4099 (54.04%) articles. After title and abstract screening, of the 4099 articles, 973 (23.74%) remained and underwent a full-text screening by 2 independent reviewers (2 from MLV, EH, MT, and Sydney Murdoch). Within this subsample of 973 articles, 949 (97.5%) were excluded because of ineligibility and 3 (0.3%) were excluded because of unclear eligibility despite multiple efforts to contact authors for clarification.

### Study and Participant Characteristics

The characteristics of the included studies are summarized in Multimedia Appendix 3 [44-64]. Funding source, outcomes, and measures are summarized in Table 1. A total of 20 interventions delivered to 2438 participants (sample size range: 47 to 361 participants per study) were examined [44-64]. The studies spanned 7 countries, including Canada, the United States, Italy, the United Kingdom, Australia, the Netherlands, and Spain. Most interventions took place in North America.

All 21 studies included in this review assessed changes in mental health conditions or concerns. The most commonly reported outcomes were anxiety, coping, depression, mental distress, and quality of life. None of the included studies reported changes in substance use or bereavement. More than half of the included studies compared interventions using inactive control, which was defined as no intervention, waitlist, or usual care [44,47,49,52,53,55-58,60,61,63]. This included 2 RCTs (2/21, 10%) that compared 2 interventions that met the inclusion criteria with each other and with inactive control [49,58]. The remaining RCTs in this review compared the intervention with active control, which was defined as any intervention that differed from the treatment and did not meet the eligibility criteria for this review [45,46,51,59,62,64]. Typical active controls were in-person interventions, unguided interventions, and one-on-one interventions. In all, 3 RCTs used both inactive and active control groups [48,50,54].

All studies reported findings by sex, and 14% (3/21) also included gender and sexual orientation (men, women, gay, and bisexual). Although sex was reported in the descriptive summaries, it was not considered during the analyses or the reporting of results, with the exception of Heckman and Carlson [48] who reported findings by male, female, and gay or bisexual categories [48]. Although the studies included in this review were unbalanced in terms of sex, with an overrepresentation of female participants in many RCTs, the findings were typically generalizable to the populations studied. We observed that interventions examining mental health among adults living with HIV had more men, whereas interventions examining mental health among adults with chronic fatigue syndrome had more women. In terms of age, this review focused on adults aged ≥18 years with a single exception. We included a study with participants aged 16-25 years, given that the mean sample age was 20 (SD 2.2) years, with 3.7% (9/244) of the participants aged <18 years [57]. No studies that met our inclusion criteria examined interventions within ethnic minority populations, and no sample was adequate for conducting stratified analysis for ethnic minority adults in this review.

All studies included in this review used self-reported symptom scores to examine the effects of an intervention. No RCT that used physician-diagnosed mental health measures was found. The specific measures used to examine changes in mental health across this review are described in Table 1. Most of the interventions sought to improve mental health among adults with a specific physical health condition, including cancer or tumors [51,59,63,64], HIV or AIDS [47-49], epilepsy [50,55,56], multiple sclerosis (MS) [44,45], bulimia [62], and chronic fatigue [46]. Several sought to improve mental health among nonprofessional caregivers [52,53,58,60,61]. Only 10% (2/20) of interventions that met the inclusion criteria sought to improve mental health among adults in the general population [54,57].

**Table 1:** Funding source, outcomes, and measures of the included studies (N=21).

Study	Funding source	Outcomes and measures					
		Anxiety	Coping	Depression	Mental distress	Quality of life	Physical health or behavior
Bogosian et al, 2015 [44]	Multiple Sclerosis Society UK (961/11)	HADS^a^	N/A^b^	HADS	GHQ^c^	EQ-5D^d^	MSIS-29^e^; FSS^f^
Cavalera et al, 2019 [45]	Fondazione Italiana Sclerosi Multipla, Italian private foundation (FISM Research Grant 2013/R/17)	HADS	N/A	HADS	N/A	MSQOL-54^g^	MOS-S^h^; MFIS^i^
Hall et al, 2017 [46]	National Institutes of Health (5R01NS055672), National Research Service Award (T32AT000051) from the National Center for Complementary and Integrative Health at the National Institutes of Health	N/A	N/A	N/A	PSS^j^	N/A	CDC-CFS^k^
Heckman et al, 2006 [47]	National Institute on Aging (R21 AG20334)	N/A	CSES^l^; WOCC^m^	GDS^n^	N/A	N/A	N/A
Heckman and Carlson, 2007 [48]	National Institute of Mental Health (RO1 MH59009)	N/A	CSES	BDI^o^	N/A	N/A	N/A
Heckman et al, 2013 [49]	Grant RO1 MH078749 from the National Institute of Mental Health and the National Institute of Nursing Research	N/A	N/A	GDS	N/A	N/A	N/A
Hum et al, 2019 [50]	EpLink: The Epilepsy Research Program of the Ontario Brain Institute	N/A	N/A	QIDS^p^ and NDDI-E^q^	N/A	WHOQOL-BREF^r^	N/A
Lepore et al, 2014 [51]	National Institutes of Health (R21CA15877)	HADS	N/A	HADS	N/A	N/A	N/A
Marziali and Donahue, 2006 [52]	National Institute of Mental Health (R34 MH092207)	N/A	N/A	N/A	RMBPC^s^	N/A	HSQ-12^t^
Park et al, 2020 [53]	Marino Health Foundation (no grant number)	PHQ-4^u^	N/A	PHQ-4	VAS^v^	N/A	N/A
Paxton et al, 2007 [54]	Australian Rotary Health Research Fund	N/A	N/A	BDI-II^w^	N/A	N/A	N/A
Thompson et al, 2010 [55]	Cooperative Agreement (U48 DP000043) through the Emory Prevention Research Center from the Centers for Disease Control and Prevention	N/A	CSES	BDI; mBDI^x^; NDDIE; PHQ-9^y^	N/A	BRFSS^z^	N/A
Thompson et al, 2015 [56]	National Institutes of Health grant (5RC1 MD004563) from the National Center for Minority Health and Health Disparities	N/A	N/A	mBDI; PHQ-9	N/A	N/A	N/A
Van der Zanden et al, 2012 [57]	ZonMw (Netherlands Organization for Health Research and Development) grant (61300036)	HADS	N/A	CES-D^aa^	N/A	N/A	N/A
Vazquez et al, 2017 [58]	Ministry of Economy and Competitiveness of Spain (2012-PN162 [PSI2012-37396])	HADS	N/A	CES-D	N/A	N/A	N/A
Vranceanu et al, 2016 [59]; Zale et al, 2018 [64]	Children’s Tumor Foundation through a clinical research grant awarded to Ana-Maria Vranceanu	GAS^ab^	MOCS-A^ac^	PHQ-9	N/A	WHOQOL-BREF	NPRS^ad^; BPI^ae^
Wakefield et al, 2016 [60]	Cancer Australia (APP1065428); the National Health and Medical Research Council of Australia (APP1067501); Cancer Institute of New South Wales (11/ECF/3-43); and Cancer Institute of New South Wales (14/ECF/1-11). The Behavioural Sciences Unit is supported by the Kids with Cancer Foundation	DASS-21^af^	N/A	DASS-21	DASS-21	QOL-FCT^ag^	N/A
Winter and Gitlin, 2007 [61]	Alzheimer’s Association grant awarded to Laura N. Gitlin, PhD	N/A	N/A	CES-D	N/A	N/A	N/A
Zernicke et al, 2014 [63]	Mind and Life Francisco J. Varela Research Award	POMS^ah^	N/A	POMS	CSOSI^ai^	N/A	N/A
Zerwas et al, 2016 [62]	National Institute of Mental Health grant (R01MH080065); Clinical Translational Science Award (UL1TR000083); and Alexander von Humboldt-Stiftung	BAI^aj^	BDI	N/A	N/A	EDQOL^ak^; SF-6D^al^	N/A

^a^HADS: Hospital Anxiety and Depression Scale.

^b^N/A: not applicable.

^c^GHQ: General Health Questionnaire.

^d^EQ-5D: EuroQol-5 Dimensions.

^e^MSIS-29: Multiple Sclerosis Impact Scale-29.

^f^FSS: Fatigue Severity Scale.

^g^MSQOL-54: Multiple Sclerosis Quality of Life-54.

^h^MOS-S: Medical Outcomes Study-Sleep.

^i^MFIS: Modified Fatigue Impact Scale.

^j^PSS: Perceived Stress Scale.

^k^CDC-CFS: Centers for Disease Control and Prevention Chronic Fatigue Syndrome Symptom Inventory.

^l^CSES: Coping Self-Efficacy Scale.

^m^WOCC: Ways of Coping Checklist.

^n^GDS: Geriatric Depression Scale.

^o^BDI: Beck Depression Inventory.

^p^QIDS: Quick Inventory of Depressive Symptomatology.

^q^NDDI-E: Neurological Disorders Depression Inventory for Epilepsy.

^r^WHOQOL-BREF: World Health Organization Quality of Life-Brief Version.

^s^RMBPC: Revised Memory and Behavior Problems Checklist.

^t^HSQ-12: Health Status Questionnaire-12.

^u^PHQ-4: Patient Health Questionnaire-4 item.

^v^VAS: Visual Analog Scale.

^w^BDI-II: Beck Depression Inventory 2.

^x^mBDI: modified Beck Depression Inventory.

^y^PHQ-9: Patient Health Questionnaire-9 item.

^z^BRFSS: Behavioral Risk Factor Surveillance System.

^aa^CES-D: Center for Epidemiological Studies-Depression.

^ab^GAS: Generalized Anxiety Scale.

^ac^MOCS-A: Measure of Current Status-Part A.

^ad^NPRS: Numeric Pain Rating Scale.

^ae^BPI: Brief Pain Inventory.

^af^DASS-21: Depression Anxiety Stress Scale-21.

^ag^QOL-FCT: Quality of Life-Family Caregiver Tool.

^ah^POMS: Profile of Mood States.

^ai^CSOSI: Calgary Symptoms of Stress Inventory.

^aj^BAI: Beck Anxiety Inventory.

^ak^EDQOL: Eating Disorder Quality of Life.

^al^SF-6D: Short Form-6 Dimensions.

Adults with a specific physical health condition were recruited through registries, community-based organizations, health centers, or clinical referrals. People living with cancer or tumors included women with stage I or II breast cancer in the past 36 months [51], patients with neurofibromatosis diagnosed by a medical professional [59,64], and those diagnosed with cancer who completed primary cancer treatment in the last 3 years [63]. Studies that examined people living with HIV or AIDS used self-reported diagnosis [47-49]. Adults with epilepsy were required to have been diagnosed with the condition for at least one year [50,55], or at least 3 months should have elapsed after the diagnosis at the time of recruitment [56]. Adults with MS were included if they had a diagnosis of primary or secondary progressive MS [44] or a diagnosis of relapsing–remitting or secondary progressive MS, as determined by a neurologist [45]. Zerwas et al [62] used the Diagnostic and Statistical Manual of Mental Disorders, Fourth Edition, to assess bulimia nervosa [62]. Hall et al [46] required participants to have a physician-determined chronic fatigue syndrome diagnosis based on the definition formulated by Fukuda et al [66].

### Intervention Content and Facilitation

Of the 20 group eHealth interventions, 6 (30%) delivered CBT [46,54,57,58,60,62], 1 (5%) was a mindfulness program tested across 2 RCTs [45,63], 4 (20%) used a combination of mindfulness and CBT [44,50,55,56], 2 (10%) were defined as resilience-based programs [53,59,64], and 6 (30%) were support groups led by a health professional [47-49,51,52,61].

In all, 38% (8/21) of RCTs examined interventions delivered by videoconference [44,45,52,53,59,60,63,64], 43% (9/21) by teleconference [46-50,55,56,58,61], and 19% (4/21) by live, synchronous chat room [51,54,57,62]. Of the 20 interventions, 95% (n=19) were fully delivered by facilitators with professional training related to the intervention (the facilitators were typically mental health or allied health professionals). The sole exception had some peer-led delivery but was included, given that the intervention had significantly more contact hours than most interventions examined, and the first 10 hours had been delivered solely by certified health professionals [52].

### Intervention Effectiveness by Outcome

#### Overview

**Figure 2:**
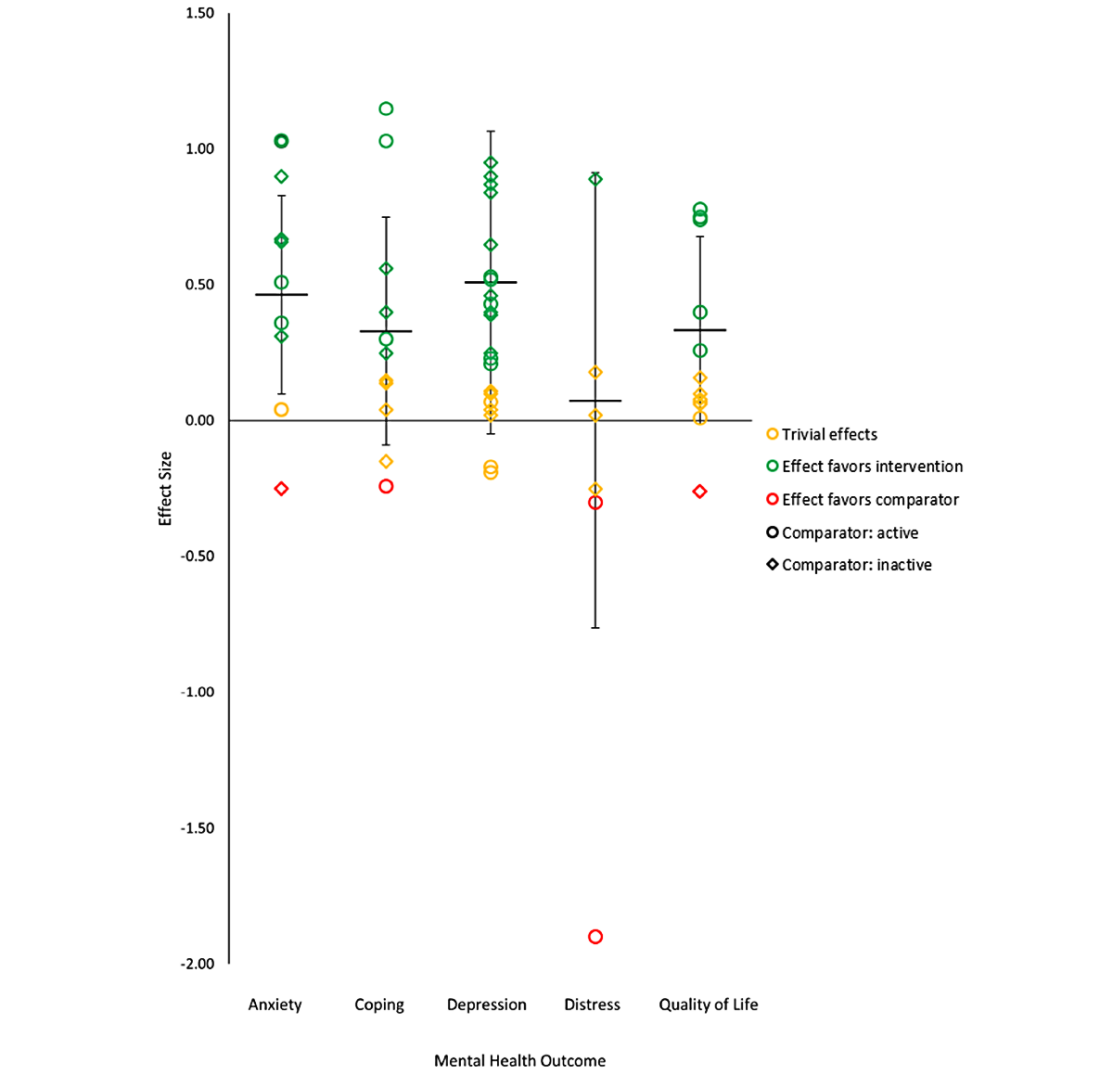
Effect sizes for mental health outcomes among the included studies compared with inactive and active controls.

Key outcomes examined across most of the RCTs were depression (17/21, 81% of the studies), anxiety (8/21, 38%), mental distress (6/21, 24%), coping (4/21, 19%), and quality of life (8/21, 38%). None of the RCTs examined substance use, addiction, or bereavement outcomes, highlighting a gap in knowledge regarding the use of live, health professional–led group eHealth interventions for these outcomes. Effect sizes and CIs were calculated for each study to allow for comparison of effects across RCTs, with results summarized in Table 2 and narratively in the next sections. In addition, Figure 2 provides a visual display of the results as recommended when a meta-analysis of effect estimates is not possible [43].

**Table 2:** Effect of eHealth interventions by outcome and comparator.

Outcome by comparator		Impact	Number of participants (studies)	Certainty of evidence (GRADE^a^)
**Anxiety**				
	Inactive control	Four studies had large to small effects, and 1 study had a trivial effect	446 (5 RCTs^b^)	Very low^c,d,e^
	Active control	Two studies had large to small effects, and 1 study had a trivial effect; 1 study reported inferior results, but effect sizes could not be calculated	380 (4 RCTs)	Very low^c,d,e,f^
**Bereavement**				
	Inactive control	No studies	0 RCTs	N/A^g^
	Active control	No studies	0 RCTs	N/A
**Coping**				
	Inactive control	One study had a small effect; and 1 study showed a trivial effect; 1 study had small to trivial effects favoring the control group	433 (3 RCTs)	Very low^c,d,e^
	Active control	One study had a large effect	63 (1 RCTs)	Very low^d,e,f,h^
**Depression**				
	Inactive control	Nine studies had large to small effects, and 2 studies had trivial effects; 1 study comparing 2 interventions found small effects in one and trivial effects in the other. The intervention was inferior to control in 1 study (small effect)	1488 (13 RCTs)	Low^c^
	Active control	Three studies had medium to small effects, and 2 studies had trivial effects	500 (5 RCTs)	Very low^c,d,e^
**Mental distress**				
	Inactive control	Four studies had large to medium effects, and 1 study had a trivial effect	268 (5 RCTs)	Very low^c,f,h,i^
	Active control	The intervention was inferior to control in 1 study (large effect)	100 (1 RCTs)	Very low^d,e,f^
**Quality of life**				
	Inactive control	One study had a small effect, and 2 studies had a trivial effect. The intervention was inferior to control in 1 study (small effect)	268 (4 RCTs)	Very low^c,d,f,j^
	Active control	Two studies had large to small effects, and 2 studies had a trivial effect	421 (4 RCTs)	Very low^c,e,i,j^
**Substance use**				
	Inactive control	No studies	0 RCTs	N/A
	Active control	No studies	0 RCTs	N/A

^a^GRADE: Grading of Recommendations Assessment, Development, and Evaluation.

^b^RCT: randomized controlled trial.

^c^Most articles were rated *high* using the Cochrane Risk-of-Bias Tool.

^d^Magnitude and direction of effect varied across studies.

^e^Variability in how the outcome is measured and the types of interventions.

^f^The total number of participants across studies was small (400 or fewer), and some studies had small improvements, whereas others had nonsignificant results likely because of a small sample size (borderline imprecision).

^g^N/A: not applicable.

^h^At least one study was rated *high*, and multiple studies rated *some concerns* overall with the Cochrane Risk-of-Bias Tool.

^i^Populations are limited to a few specific conditions and disorders or by sex, which limits generalizability.

^j^The total number of participants across studies was >400, but some studies found no effect (borderline imprecision).

#### Anxiety

In all, 38% (8/21) of RCTs examined intervention effectiveness for anxiety, which was most commonly measured using the Hospital Anxiety and Depression Scale (n=5 studies). The Patient Health Questionnaire (n=1), the Generalized Anxiety Scale (n=1), the Depression Anxiety Stress Scale Short Form (n=1), the Beck Anxiety Inventory (n=1), and the Profile of Mood States (n=1) were also used. Compared with inactive control, health professional–led group eHealth interventions delivered live to adults had large to small effects on anxiety across 4 studies [44,53,57,63] and a trivial effect in 1 study [60]. Compared with active control, the interventions had large to small effects on anxiety in 2 studies [45,59] and a trivial effect in 1 study [62]. Active control was superior to intervention in 1 study; however, we were unable to calculate the effect size using the available data [62]. Taken together, the interventions had a medium effect on reducing anxiety compared with inactive (Cohen *d*=0.57, 95% CI 0.17-0.90) or active control (Cohen *d*=0.48, 95% CI 0.15-0.81).

#### Coping

A total of 24% (5/21) of studies examined intervention effectiveness for perceived coping. Most of them used the Coping Self-Efficacy Scale (n=3 studies). The Beck Depression Inventory, Measure of Current Status-Part A, and the Ways of Coping Checklist were also used. Compared with inactive control, interventions had a small to trivial effect on coping across 2 studies [47,56]. An additional study compared 2 eligible interventions with inactive control; the effect sizes suggest that the interventions were inferior [48]. Compared with active control, an eligible intervention had a large effect on improving coping (Cohen *d*=1.15, 95% CI values could not be computed) [64]. On the basis of these results, we conclude that more studies are needed before results can be pooled and conclusions drawn about the effectiveness of live health professional–led group eHealth interventions on coping.

#### Depression

Overall, 81% (17/21) of RCTs examined intervention effectiveness for depression, which was measured using well-recognized instruments for this construct, including the Beck Depression Inventory (n=4 studies), the Hospital Anxiety and Depression Scale (n=3), the Patient Health Questionnaire (n=3), or the Centre of Epidemiological Studies Depression Scale (n=3). Compared with inactive control, 9 interventions had large to small effects on depression [44,50,53,56-58,60,61,63], 1 study had small to trivial effects [49], and 1 study had a trivial effect [47]. Compared with active control, 3 studies had medium to small effects on depression (compared with psychoeducation control) [45,50,59]. However, the intervention was inferior to in-person delivery of the same intervention for depression in 2 studies [54,62]. Taken together, live health professional–led group eHealth interventions had medium effects on adult depression compared with inactive control (Cohen *d*=0.61, 95% CI 0.33-0.89) and small effects on adult depression compared with active control (Cohen *d*=0.21, 95% CI –0.19 to 0.53).

#### Mental Distress

In all, 24% (5/21) of RCTs examined intervention effectiveness for mental distress, which was examined using a different measure in each of the 5 studies that assessed it. The measures used were the Calgary Symptoms of Stress Inventory, General Health Questionnaire, Perceived Stress Scale, Visual Analog Scale, Revised Memory and Behavior Problems Checklist, and the Depression Anxiety Stress Scale-21 (Table 1). Compared with inactive control, interventions had large to medium effects on mental distress in 4 studies [44,52,53,63] and no effect on mental distress in 1 study [60]. Compared with active control, the intervention was inferior in 1 study compared with in-person delivery (Cohen *d*=–1.90) [46]. When effect sizes were pooled, the eligible interventions yielded a moderate effect on mental distress compared with inactive control (Cohen *d*=0.72, 95% CI –0.04 to 0.85) across 5 studies but inferior to the same intervention delivered in person. Thus, although live health professional–led group eHealth interventions may be effective for addressing mental distress among adults compared with inactive control, more research is needed to determine whether it is effective compared with the same intervention delivered in person.

#### Quality of Life

This review did not systematically search for quality-of-life outcomes. However, 38% (8/21) of studies that met the inclusion criteria reported intervention effectiveness for this outcome. We have summarized this information, noting that this may not comprehensively summarize all the RCTs that assessed the effect of live eHealth professional–led group interventions on quality of life. As shown in Table 1, quality of life was measured using a variety of instruments. Compared with inactive control, the eligible interventions had a small effect on quality of life in 1 study [44], had a trivial effect in 2 studies [55,56], and were inferior to control in 1 study [60]. Compared with active control, the eligible interventions had large to small effects on quality of life in 2 studies [45,59], and a trivial effect on quality of life in 2 studies [50,62]. When the findings were pooled, we found that live eHealth professional–led group interventions had trivial effects on quality of life compared with inactive control (Cohen *d*=0.07, 95% CI –0.26 to 0.32) and active control (Cohen *d*=0.19, 95% CI 0.07-0.76).

#### Physical and Behavioral Health

This review did not search for physical or behavioral health outcomes beyond substance use. Given that 24% (5/21) of RCTs selected for this review reported these outcomes, we have summarized the evidence. As shown in Table 1, sleep, fatigue, pain, MS, and general health were examined using a variety of measures. An eligible intervention resulted in significant reductions in fatigue compared with inactive control [44]. However, the results were mixed when compared with active control, with a live health professional–led group eHealth intervention having no effect on fatigue [37] and a second intervention proving inferior to this comparator [38]. For sleep, a study reported significant improvements in sleep quality and sleep quantity compared with active control [37]. For pain, an eligible intervention had a significant impact on pain compared with inactive control [44]. Compared with active control, an intervention reduced pain intensity, but this change did not significantly differ from control at posttest assessment [50]. A study found that a live health professional–led group eHealth intervention significantly reduced the perceived burden of MS on participants’ lives compared with inactive control [44]. Finally, a study found that an eligible intervention had no impact on perceived general health compared with no intervention [52].

### Effectiveness by Intervention Delivery Platform

#### Videoconferencing

A total of 38% (8/21) of RCTs examined interventions delivered by videoconference, all of which reported large to small effects on improved mental health compared with inactive [44,52,53,60,63] and active control [45,59,64]. When effect sizes were pooled across comparators, the eligible interventions delivered by videoconferencing had a medium effect on adult anxiety (Cohen *d*=0.60, 95% CI 0.17-1.03), depression (Cohen *d*=0.60, 95% CI 0.38-0.90), and mental distress (Cohen *d*=0.72, 95% CI 0.18-1.03) and a small effect on quality of life (Cohen *d*=0.43, 95% CI –0.03 to 0.99).

#### Teleconferencing

In all, 43% (9/21) of RCTs examined interventions delivered by teleconference. Compared with inactive control, 6 resulted in large to small improvements in mental health [49,50,55,56,58,61], 1 had a trivial effect [47], and 1 intervention was inferior [48]. Compared with active control, an eligible intervention delivered by teleconference had a small effect on improving mental health in 1 study [40] but was inferior at improving mental health across 2 studies [46,48]. When effect sizes were pooled, teleconference interventions had a medium effect on depression (Cohen *d*=0.50, 95% CI 0.16-1.08), had a trivial effect on coping (Cohen *d*=0.04, 95% CI –0.03 to 0.10) and quality of life (Cohen *d*=0.09, 95% CI 0.06-0.16), and were inferior to control for mental distress (Cohen *d*=–1.90, 95% CI –0.24 to 0.05).

#### Live Chat Room

Live, synchronous chat rooms were used to deliver interventions across 19% (4/21) of interventions in this review. Compared with inactive control, 2 studies reported large to medium effects on depression and anxiety [54,57]. Compared with active control, 1 study found a trivial effect and 2 studies found that active control was superior (the same intervention delivered face to face) [51,54,62]. When effect sizes were pooled across comparators, interventions delivered by live chat room had a small effect on adult anxiety (Cohen *d*=0.35, 95% CI –0.08 to 0.78) and depression (Cohen *d*=0.24, 95% CI –0.11 to 0.59).

### Effectiveness by Intervention Intensity

#### Contact Hours: <8

Given that only 10% (2/21) of RCTs examined interventions with less than 8 group-based contact hours, we did not pool effect estimates for interventions of this length. Both interventions sought to improve mental health among nonprofessional caregivers. The first intervention had a large effect on depression compared with inactive control and trivial effects compared with active control [58]. The second intervention had small effects on depression, anxiety, and mental distress among parents caring for children with cancer compared with waitlist control [60].

#### Contact Hours: 8-12

The majority of RCTs (11/21, 52%) examined interventions that had 8-12 group-based contact hours [44,48,50,51,53-57,59,61]. Compared with inactive control, 6 found the interventions superior at improving mental health [44,53-57] and 1 found a trivial effect compared with usual care [48]. Compared with active control, 3 interventions of this length were superior at improving mental health [50,59,64], whereas 2 found that active control was superior [48,54]. When effect sizes were pooled, interventions with 8-12 contact hours had a medium effect on adult anxiety (Cohen *d*=0.57, 95% CI 0.04-1.03) and mental distress (Cohen *d*=0.75, 95% CI 0.05-0.44) and a small effect on coping (Cohen *d*=0.35, 95% CI –0.09 to 0.54) and depression (Cohen *d*=0.40, 95% CI 0.21-0.60).

#### Contact Hours: >12

In all, 38% (8/21) of RCTs examined eligible interventions with more than 12 group-based contact hours [45-47,49,52,61-63]. A total 22 contact hours were provided by 1 study, with the first 10 hours delivered by a health professional and the last 12 hours by peer-led support. We have included this study in the >12-hour category, given that the outcomes were only measured at the end of 22 contact hours [52]. Compared with inactive control, 4 studies found that interventions of this length had large to small effects on mental health outcomes [49,52,61,63] and 1 study found a trivial effect [47]. Compared with active control, 1 study found medium to small effects on mental health [45], 1 study found a trivial effect compared with in-person delivery of the same intervention [62], and 1 study found that in-person delivery was superior [46]. When effect sizes were pooled, interventions with more than 12 contact hours had a medium effect on anxiety (Cohen *d*=0.63, 95% CI 0.26-1.00), a small effect on depression (Cohen *d*=0.33, 95% CI 0.02-0.90), and a trivial effect on coping (Cohen *d*=0.07, 95% CI 0.09-0.21) and mental distress (Cohen *d*=0.01, 95% CI –0.64 to 0.67).

### Nonprofessional Caregivers

**Figure 3:**
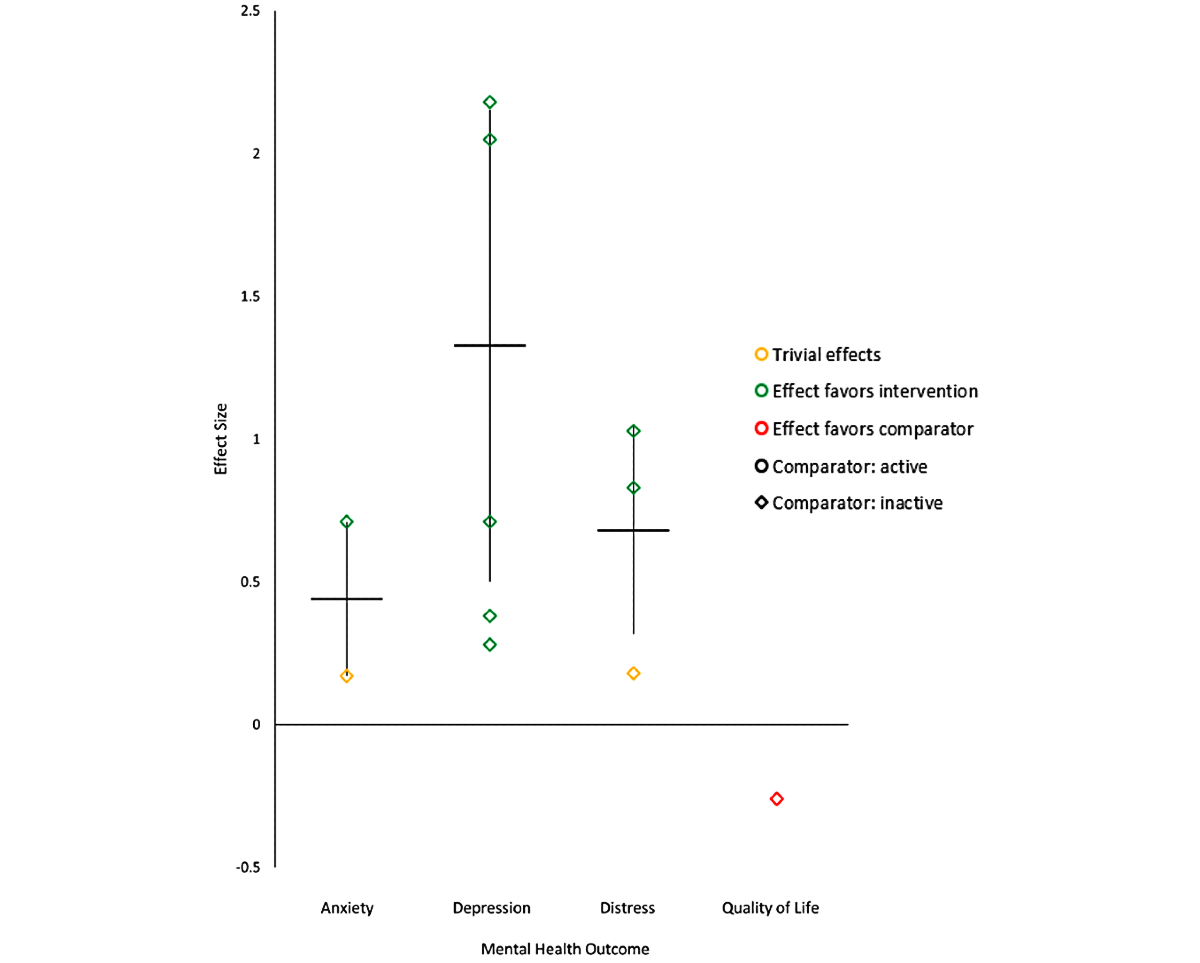
Effect sizes for mental health outcomes among caregivers compared with inactive control.

Almost one quarter of RCTs in this review (5/21, 24%) assessed interventions designed to improve nonprofessional caregiver mental health. These findings were pooled into our overall results. We also present the results for this subgroup separately in Figure 3. To summarize these results, the eligible interventions had beneficial effects on caregiver anxiety [53,60], depression [53,58,60,61], and mental distress [52,53]. Compared with inactive control, 1 study found trivial effects on anxiety [60]. None of the RCTs identified in this review focused on professional caregivers (ie, those trained and paid to provide care), which may be a gap to address in future studies.

### Risk of Bias

**Figure 4:**
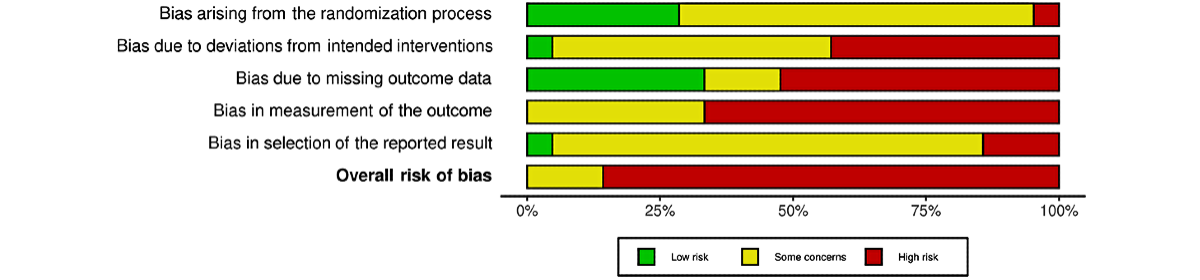
Risk-of-bias graph: review of authors’ judgments about each risk-of-bias item presented as percentages across all included studies.

**Figure 5:**
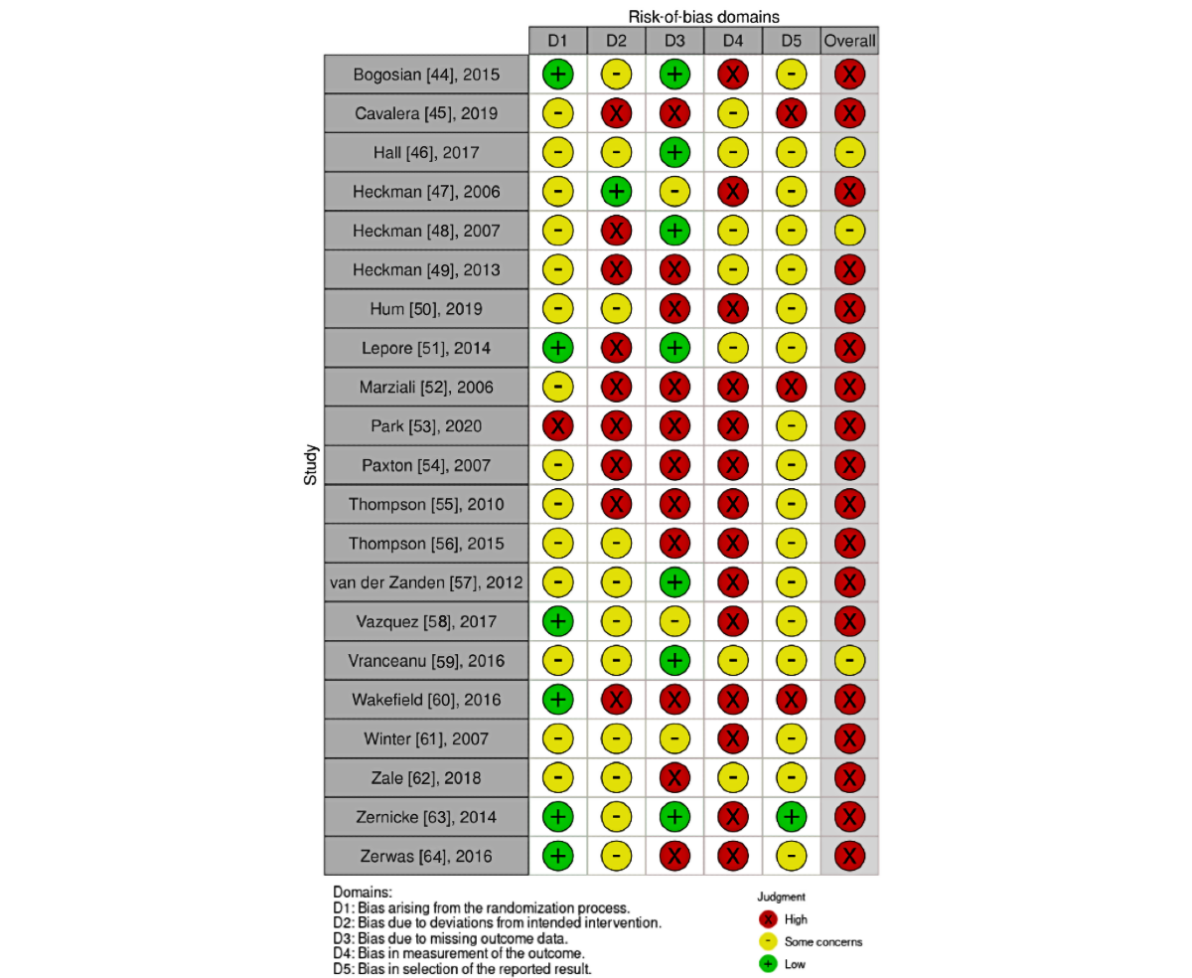
Risk-of-bias summary: review of authors’ judgments about each risk-of-bias item for each included study [44-64].

Risk-of-bias assessments are presented in Figure 4 and Figure 5 [44-64], with more detailed information presented in Multimedia Appendix 4 [44-64]. Most of the studies (19/21, 91%) received a high risk-of-bias rating, given that the behavioral interventions tested would be difficult to conceal. In all, 19% (4/21) of the RCTs examined interventions that blinded study staff during the data cleaning and analysis portions of the studies [44,51,60,63]. For example, an intervention blinded participants by telling them that they were testing 2 stress management interventions without revealing which was the treatment condition. We included 2 RCTs reporting outcomes from this blinded intervention in this review [59,64]. Risk-of-bias ratings were also affected by the measurement of outcomes, which were typically reported using surveys with participants rather than through independent assessors [67]. Finally, risk-of-bias ratings were affected by missing information about allocation concealment in 67% (14/21) of studies [45-49,52-57,59,61,64] and missing information about the randomization process in 33% (7/21) of studies [46,48,50,52,55,56,61].

The quality of evidence in this review was typically rated very low because of small sample sizes. The exception was depression compared with inactive control, which was rated low given the larger number of studies, larger samples, and medium to large effects (Table 2). For caregiver populations, all outcomes were rated as having very low certainty because of small sample sizes across included studies.

### Knowledge Gaps

No eligible RCT that examined impacts on substance use or bereavement was found despite our use of search terms to specifically identify studies that had examined these outcomes. In addition, none of the studies identified in this review reported that they had encouraged physical activity as a way to address or improve mental health as part of their intervention. Finally, no eligible intervention identified in this review focused on professional caregivers or health professionals.

## Discussion

### Principal Findings

This systematic review assessed experimental evidence for the effectiveness of live health professional–led group eHealth interventions on mental health, substance use, or bereavement among community-dwelling adults. A total of 20 unique interventions met the inclusion criteria for this review across 21 RCTs. Evidence was summarized for 2438 adults in 7 countries. All participants were community-dwelling; most had underlying physical health conditions and were taking part in the intervention to address mental health concerns related to their health.

The interventions identified in this review had the strongest and most consistent effects on reducing anxiety and depression. Medium effects on anxiety were observed compared with inactive or active control across studies. This is not unexpected, given that eHealth interventions may be particularly effective for anxiety disorders such as agoraphobia that have symptoms exacerbated by travel [11]. For depression, the interventions had medium to small effects across studies compared with inactive and active controls. These findings build on a pair of recent systematic reviews that concluded that live health professional–led videoconference interventions are effective at improving adult anxiety and depression when delivered *one-on-one* [11,12]. Extending these findings to groups in this review is encouraging, given that group delivery of live eHealth interventions could expand public access to mental health professionals in cost- and time-effective ways, both during and after the pandemic. That said, the effectiveness of the interventions examined in this review for mental distress and coping were unclear across studies, highlighting the need for more research on these outcomes.

No RCT was found that examined intervention impacts on substance use or bereavement, highlighting notable gaps in the evidence base. More broadly, systematic reviews of RCTs have shown that internet- and computer-based interventions are effective at reducing grief, as well as cannabis and illicit drug use among adults, although the number of RCTs used to make these assessments remains small [6,30,68]. Thus, it is plausible that live, eHealth group interventions led by health professionals could also be effective at addressing these outcomes. Determining the extent to which this is the case and the superiority of such interventions for these outcomes compared with in-person, asynchronous, or one-on-one eHealth interventions requires further RCT research.

When the results were examined by eHealth platform, interventions delivered by group videoconference had the most robust impacts on adult mental health. Medium effects on reduced anxiety, depression, and mental distress were observed across videoconference interventions, in addition to a small effect on improved quality of life. Eligible teleconference interventions had medium effects on depression. However, trivial effects were observed for improvements in perceived coping and quality of life, and a study found that an intervention delivered by teleconference was inferior to control for mental distress. Although only a few RCTs (n=4) examined interventions delivered using live web-based chat, small but encouraging positive effects were observed for adult anxiety and depression.

In terms of intervention length, 90% (19/21) of the RCTs in this review examined interventions that offered ≥8 live, group contact hours led by a health professional. Interventions with 8-12 contact hours had medium effects on anxiety and mental distress and small effects on depression and coping across 11 RCTs. Interventions with >12 contact hours were assessed by 38% (8/21) of RCTs and also had a medium effect on anxiety and a small effect on depression. This is surprising, given that research suggests that more time is generally better to build a strong therapeutic bond in a digital intervention [15,69]. Building from these results, a hypothesis that could be tested in future studies is whether working with clients in groups using eHealth platforms encourages therapeutic bonds to be built more efficiently compared with more standard one-on-one eHealth therapies. The findings of this review also highlight the need for more research generally, given that the heterogenous nature of the interventions identified, the risk of bias, and the quality of evidence available to date make it difficult to ascertain whether the observed effects were due to intervention length or other factors.

The conflictive results observed in this review may also be due to differences in the target populations examined. Many participants in this review had underlying health conditions, which suggests that some may have been receiving concomitant treatments as part of their usual care that could have influenced outcomes. It may also be that chronic disease or pain among participants in some studies influenced the extent of their engagement. Inconsistent results may also be due to differences in the therapeutic methods used (eg, mindfulness-based cognitive therapy, CBT, and mindfulness-based stress reduction). Of note, we were not able to draw conclusions on the effectiveness of specific therapeutic methods because few studies used the same method. Differences in the types of control groups used across studies may have also influenced the discrepant results. For example, when an active intervention was found to be inferior in this review, it was often in studies that compared it with an active control that was the same intervention delivered face to face. Finally, many of the RCTs included in this review were described as pilot studies (9/21, 43%) and may not have had the study power to draw reliable conclusions.

### Limitations

This review was limited to studies published in English or French. The search did not include physical or behavioral health outcomes or gray literature. Clinical experts (eg, psychiatrists) were not involved in the search. All measures used to assess changes in mental health in this review were self-report, and the overall number of studies summarized was small. The nature of the interventions under study (ie, behavioral rather than pharmaceutical) meant that participants were usually unblinded. For these reasons, no study captured in this review was rated lower than *moderate* for risk of bias. Intervention adherence and attendance were not consistently reported across the RCTs selected for this review and could not be assessed. All participants were community-dwelling, and most had underlying chronic health problems. Given that chronic health conditions are common among adults, this may not hamper generalizability, although generalizability to adults without underlying health conditions should be made with caution [57,58]. We also note that the intensity of usual care varied across the RCTs in this review, raising concerns about the interpretation and generalizability of results. We also note that a study identified in this review included 9 participants who were aged 16-17 years. The reason for this exception to the inclusion criteria is that the proportion of the sample who were not aged ≥18 years was extremely small (9/244, 3.8%) and likely inconsequential to the results, given that the remaining bulk of the sample (235/244, 96.2% of the participants) were aged ≥18 years. Thus, we decided that removing this particular study would be a greater violation of our inclusion criteria than including it would be a violation of our exclusion criteria. This study had one of the largest sample sizes of adults who met all inclusion criteria; thus, it was our opinion that removing it would result in a systematic review that did not reflect the published studies we sought to summarize. We can confirm that no other study in the pool of papers selected from our original literature search that met all other inclusion criteria for this review had a mixed adolescent or adult sample. Finally, few studies selected for this review included a follow-up period of ≥6 months. As a result, the longer-term impacts of live health professional–led group eHealth interventions on adult mental health remains unclear.

### Conclusion

Live eHealth group interventions led by health professionals can foster moderate improvements in anxiety, and moderate to small improvements in depression among community-dwelling adults, particularly those delivered by videoconference and those providing 8-12 hours of synchronous engagement. This review highlights a need for experimental research to understand the long-term impacts of these interventions and whether they may be effective for adult substance use and bereavement.

## Appendix

Multimedia Appendix 1 MEDLINE search strategy.

Multimedia Appendix 2 Effect size calculation summary.

Multimedia Appendix 3 Characteristics of included studies (N=21).

Multimedia Appendix 4 Risk-of-bias screening results.

## Abbreviations

CBT: cognitive behavioral therapy
MS: multiple sclerosis
PRISMA: Preferred Reporting Items for Systematic Reviews and Meta-Analyses
RCT: randomized controlled trial
