# Supplementary material for: Effectiveness of Live Health Professional–Led Group eHealth Interventions for Adult Mental Health: Systematic Review of Randomized Controlled Trials
Source: J Med Internet Res. 2022 Jan 11;24(1):e27939. doi: 10.2196/27939 (PMC8790691; doi:10.2196/27939)
Supplement: Multimedia Appendix 1 [file jmir_v24i1e27939_app1.pdf]

Appendix 1. MEDLINE search strategy

1. Mental Health/ [MeSH]
2. exp Mental Disorders/ [MeSH]
3. exp Behavioral Symptoms/ or exp Bereavement/
4. (((mental* or psychological*) adj3 (health* or well* or disorder* or ill*)) or anxi* or depress* or neuros* or psychiatric or stress* or distress* or emotion* or aggress* or trauma* or suicid* or bereav* or grief or griev* or mourn* or addict* or alcoholism or ((substance* or drug* or alcohol*) adj3 (us* or misus* or abus* or dependen*))).mp.
5. or/1-4
6. exp Videoconferencing/ [MeSH]
7. exp Telemedicine/ {MeSH]
8. exp Internet/ {MeSH] or exp Telephone/ [MeSH]
9. (telehealth or telemedicine or ehealth or video*).mp.
10. (Skype or Facetime or Zoom or Google+Hangouts).mp.
11. (internet or web or online or telephon* or phone or phoning or phones or phoned or SMS or text messag* or texting or texted).mp.
12. (distance or remote).mp.
13. or/6-12
14. exp Counseling/ [MeSH] or exp Psychotherapy/ [MeSH] or exp Nursing/ [MeSH] or exp Social Work/ [MeSH] or Yoga/ [MeSH] or Meditation/ [MeSH] or Mindfulness/ [MeSH]
15. (counsel* or motivational interview* or coach* or psychotherap* or social work* or nurs* or kinesiolog* or yoga or meditat* or mindfulness).mp.
16. or/14-15
17. 13 and 16
18. Distance Counseling/ [MeSH]
19. (e-therap* or etherap* or e-counsel* or ecounsel* or telepsycholog* or "tele-mental health" or e-coach* or ecoach*).mp.
20. or/17-19
21. group*.mp.
22. exp Adult/ [MeSH]
23. adult*.mp.
24. or/22-23
25. exp Randomized Controlled Trial/ [MeSH]
26. (randomi* or randomly).mp, pt.
27. RCT*.mp
28. or/22-24
29. 5 and 20 and 21 and 24 and 28
30. limit 29 to (yr="2005 -Current" and (english or french))
