# Supplementary material for: Effectiveness of Live Health Professional–Led Group eHealth Interventions for Adult Mental Health: Systematic Review of Randomized Controlled Trials
Source: J Med Internet Res. 2022 Jan 11;24(1):e27939. doi: 10.2196/27939 (PMC8790691; doi:10.2196/27939)
Supplement: Multimedia Appendix 2 [file jmir_v24i1e27939_app2.pdf]

Appendix 2. Effect size calculation summary

Lead Author (Year)	Calculation of Cohen's *d*				
	**Conceptual data (int vs. con)**	**Numerical Data**	**Formula**	**Int vs. Passive Con**	**Int vs. Active Con**
Bogosian (2015)	Post-intervention outcome scores (means ± SD)	N = 36, int (n = 17) vs con (n = 19); *Distress*: int (11.43±4.55) vs con (14.87±5.94); *Depression*: int (5.12±3.2) vs con (7.63±3.96); *Anxiety*: int (5.48±2.75) vs con (6.58±3.42);	$d = (M_2 - M_1)/SD_{pooled}$ $SD_{pooled} = \sqrt{((SD_1^2 + SD_2^2)/2)}$	Depression: d = 0.65[c] Distress: d = 0.67[c] Anxiety: d = 0.40[b]	N/A
Cavalera (2019)	Post-intervention between group changes	N = 139, int (n = 54) vs con (n = 67); *Depression*: $F(1,111) = 5.56$, $p = 0.020$; *Anxiety*: $F(1,111) = 3.96$, $p = 0.049$; *QOL*: $F(1,110) = 4.68$, $p = 0.033$	$d = \sqrt{(F((n_t+n_c)/n_t n_c)((n_t+n_c)/(n_t+n_c-2)))}$	N/A	Depression: d = 0.43[b] Anxiety: d = 0.36[b] QOL: d = 0.40[b]
Hall (2017)	Post-intervention outcome scores (means ± SD)	N = 100, int (n = 56) vs con (n = 44); *Distress*: int (29.98±1.35) vs con (27.11±1.65)	$d = (M_2 - M_1)/SD_{pooled}$ $SD_{pooled} = \sqrt{((SD_1^2 + SD_2^2)/2)}$	N/A	Distress: d = -1.9[d]
Heckman (2006)	Post-intervention outcome scores (means ± SD)	N = 90, int (n = 44) vs con (n = 46); *Depression:* int (14.70±9.10) vs con (14.40±7.60); *Engagement Coping*: int (2.24±0.56) vs con (2.28±0.53); *Avoidant Coping*: int (1.91±0.55) vs con (2.03±0.42); *Distress:* int (40.10±16.80) vs con (39.80±13.10)	$d = (M_2 - M_1)/SD_{pooled}$ $SD_{pooled} = \sqrt{((SD_1^2 + SD_2^2)/2)}$	Depression: d = 0.04[a] Coping: $d_{engagement}$ = 0.07[a]; $d_{avoidant}$ = 0.25[b] Distress: d = 0.02[a]	N/A

Heckman (2007)	Post-intervention outcome scores (means ± SE)	N = 299, int (n = 108) vs passive con (n = 107) vs active con (n = 84); *Depression:* int (20.23±0.67) vs passive con (20.73 ± 0.66) vs active con (19.09±0.75)	SD = SE * √N<br>$d = (X_1 - X_2)/SD_{pooled}$	Depression: d = 0.10 [a]<br>Coping: d = -0.15[a]<br>Distress: d = -0.25[b]	Depression: d = -0.17[a]<br>Coping: d = -0.24[b]<br>Distress: d = -0.30[b]
Heckman (2013)	Post-intervention outcome scores (means ± SE)	N = 361, Int_1 (n = 118, 14.36±0.47) vs Int_2 (n = 122, 11.94±0.47) vs con (n = 121, 14.34±0.47)	SD = SE * √N<br>$d = (X_1 - X_2)/SD_{pooled}$	Int_1 - Depression: d = 0.02[a]<br>Int_2 - Depression: d = 0.46[b]	N/A
Hum (2019)[c]	Post intervention QIDS scores (means ± SE) between int, passive con, and active con. NDDIE & QOL scores reported post-intervention once passive con (wait list) participants had completed the intervention.	*Depression (QIDS):* N = 55, int (n= 20; 9.55±1.1), active con (n = 24, 10.63±1.0), passive con (n = 11; 10.73±1.5)<br>*Depression (NDDIE):* N = 42, int (n=19; 16.05±0.8), active con (n=23; 15.35±0.7); *QOL:* N = 42, int (n=19; 50.66±3.9), active con (n=23; 49.46±3.5)	SD = SE * √N<br>$d = (X_1 - X_2)/SD_{pooled}$	Depression: d_{QIDS} = 0.25[b]	Depression: d_{QIDS} = 0.23[b]; d_{NDDIE} = 0.21[b]<br>QOL: d = 0.07[a]
Lepore (2014)	Changes in depression and anxiety scores	N = 183 with int (n=88), con (n=95); *Depression:* unstandardized regression coefficient (B) = 0.65 (95%CI = 0.48, 0.83); *Anxiety:* unstandardized regression coefficient (B) = 0.62 (95%CI = 0.45, 0.90)	SD = √N * (upper limit of 95% CI - lower limit of 95% CI / 3.92)<br>X1 – X2 ≈ B	N/A	Depression: d = 0.52[c]<br>Anxiety: d = 0.51[c]
Marziali (2006)	Post-intervention between group changes	N = 38, int (n = 23) vs con (n = 15); $F(1,37) = 9.68$, $p < 0.004$	$d = \sqrt{(F((n_t+n_c)/n_t n_c)((n_t+n_c)/(n_t+n_c-2)))}$	Distress: d = 1.03[d]	N/A

Park (2020)	Post-intervention outcome scores (means ± SD)	N = 53, int (n = 31) vs con (n = 22); *Depression/Anxiety*: int (2.6±1.6) vs con (3.6±2.9); *Distress*: int (3.8±1.8) vs con (5.6±1.8)	$d = (M_2 - M_1)/SD_{pooled}$ $SD_{pooled} = \sqrt{((SD_1^2 + SD_2^2)/2)}$	Depression: d = 0.71[c] Anxiety: d = 0.71[c] Distress: d = 0.83[d]	N/A
Paxton (2007)	Post-intervention intention to treat outcome scores (means ± SD)	N = 116, int (n = 37) vs active con (n = 42) vs passive con (n = 37); *Depression*: int (16.5±11.7) vs active con (14.2±12.0) vs passive con (18.4±11.0)	$d = (M_2 - M_1)/SD_{pooled}$ $SD_{pooled} = \sqrt{((SD_1^2 + SD_2^2)/2)}$	Depression: d = 0.18[a]	Depression: d = -0.19[a]
Thompson (2010)	Post-intervention between group changes	N = 40, int (n = 26) vs con (n = 27); *Depression* (BDI): F(1,37) = 11.99, p = 0.001; *Depression* (mBDI): F(1,37) = 10.08, p = 0.003; *Physical QOL*: F(1,37) = 0.05, p = 0.486, *Mental QOL:* F(1,37) = 0.09, p = 0.767; *Coping*: F(1,37) = 2.14, p = 0.152	$d = \sqrt{(F((n_t+n_c)/n_t n_c)((n_t+n_c)/(n_t+n_c-2)))}$	Depression: d_{BDI} = 0.95[d]; d_{mBDI} = 0.87[d] QOL: $d_{physical}$ = 0.06[a]; d_{mental} = 0.08[a] Coping: d = 0.40[b]	N/A
Thompson (2015)	Post-intervention between group changes	N = 118, int (n = 62) vs con (n = 56); *Depression (mBDI)*: F(1,106) = 4.67, p = 0.033; *Depression (BDI):* F(1,106) = 4.50, p = 0.36; *Depression (NDDIE):* F(1,106) = 0.35, p = 0.555; *Anxiety*: F(1, 106) = 2.75, p = 0.10; *Physical QOL*: F(1, 105) = 0.74, p = 0.392; *Mental QOL*: F(1,105) = 0.28, p = 0.600	$d = \sqrt{(F((n_t+n_c)/n_t n_c)((n_t+n_c)/(n_t+n_c-2)))}$	Depression: d_{BDI} = 0.39[b]; d_{mBDI} = 0.40[b]; d_{NDDIE} = 0.11[a] Anxiety: d = 0.31[b] QOL: $d_{physical}$ = 0.16[a]; d_{mental} = 0.10[a] Coping: d = 0.15[a]	N/A
Van der Zanden (2012)	Post-intervention outcome scores (means ± SD), missing values not replaced	N = 194, int (n = 96) vs con (n = 98); *Depression*: int (19.1±10.7) vs con (26.7 ± 9.4); *Anxiety*: int (7.8±4.2) vs con (10.2±3.5)	$d = (M_2 - M_1)/SD_{pooled}$ $SD_{pooled} = \sqrt{((SD_1^2 + SD_2^2)/2)}$	Depression: d = 0.84[d] Anxiety: d = 0.66[c]	N/A

Vazquez (2017)	Post-intervention outcome scores between two intervention groups and contol (means ± SD)	N = 61, Int_1 (n = 20; 10± 5.7) vs Int_2 (n = 22, 10.9±5.6) vs con (n = 19, 23.8 ± 6.9)	d = $(M_2-M_1)/SD_{pooled}$ $SD_{pooled} = \sqrt{((SD_1^2 + SD_2^2)/2)}$	Int_1 - Depression: d = 2.18[d] Int_2 - Depression: d = 2.05[d]	N/A
Vranceanu (2016)	Unadjusted post-intervention outcome scores (means ± SD)	N = 63, int (n = 32) vs con (n = 31); *Depression*: int (13.56±5.13) vs con (16.35±5.26); *Anxiety*: int (10.42 ± 3.15) vs con (13.68 ± 3.16); *Physical QOL*: int (76.34±19.00) vs con (61.06±22.21); *Psychological QOL*: int (67.72±17.87) vs con (53.36±20.16); *Social QOL*: int (63.28±22.78) vs con (57.53±22.18); *Environment QOL*: int (79.68±17.87) vs con (65.42±18.66)	d = $(M_2-M_1)/SD_{pooled}$ $SD_{pooled} = \sqrt{((SD_1^2 + SD_2^2)/2)}$	N/A	Depression: d = 0.53[c] Anxiety: d = 1.03[d] QOL: $d_{physical}$ = 0.74[c]; d_{psych} = 0.75[c]; d_{social} = 0.26[b]; $d_{environment}$ = 0.78[c]
Wakefield (2016)	Post-intervention outcome scores (means ± SD)	N = 35, Int (n = 19) vs con (n = 16); *Depression:* int (6.95±5.90) vs con (9.38±6.96); *Anxiety:* int (5.47±7.24) vs con (6.80±8.31); *QOL:* int (24.52±2.80) vs con (23.51±4.69); *Distress:* int (15.11±7.89) vs con (16.75±10.01)	d = $(M_2-M_1)/SD_{pooled}$ $SD_{pooled} = \sqrt{((SD_1^2 + SD_2^2)/2)}$	Depression: d = 0.38[b] Anxiety: d = 0.17[a] QOL: d = -0.26[b] Distress: d = 0.18[a]	N/A
Winter (2007)	Post-intervention main effects	N = 94, int (n = 58) vs con (n = 45); F = 4.58, p = 0.121	d = $(M_2-M_1)/SD_{pooled}$ $SD_{pooled} = \sqrt{((SD_1^2 + SD_2^2)/2)}$	Depression: d = 0.28[b]	N/A
Zale (2018)	Post intervention outcome scores (means ± SD)	N = 63, int (n = 32) vs con (n = 31); *Coping*: int (32.66±9.49) vs con (20.06±12.23)	d = $(M_2-M_1)/SD_{pooled}$ $SD_{pooled} = \sqrt{((SD_1^2 + SD_2^2)/2)}$	N/A	Coping: d = 1.15[d]

Zernicke (2014)	Post-intervention outcome scores (means±SD)	N = 62, int (n = 30) vs con (n=32); *POMS*: int (18.31±4.10) vs con (37.21±3.55); *Distress*: int (40.29±3.49) vs con (56.12±3.02)	d = $(M_2-M_1)/SD_{pooled}$<br>$SD_{pooled} = \sqrt{((SD_1^2 + SD_2^2)/2)}$	Depression/Anxiety (POMS): d = 0.90[d]<br>Distress: d = 0.89[d]	N/A
Zerwas (2016)	Post-intervention effect sizes (Cohen's d)	N = 179; *Depression*: d = 0.07; *Anxiety*: d = 0.04; *QOL*: d = 0.01	N/A	N/A	Depression: d = 0.07[a]<br>Anxiety: d = 0.04[a]<br>QOL: d = 0.01[a]
