# Supplementary material for: Effectiveness of Live Health Professional–Led Group eHealth Interventions for Adult Mental Health: Systematic Review of Randomized Controlled Trials
Source: J Med Internet Res. 2022 Jan 11;24(1):e27939. doi: 10.2196/27939 (PMC8790691; doi:10.2196/27939)
Supplement: Multimedia Appendix 3 [file jmir_v24i1e27939_app3.pdf]

Appendix 3. Characteristics of included studies (N=21)

Study	Aim	Intervention				Comparison			
		Participants, n (% female); age (years; mean, SD)	Details	Group size	Weeks (contact hours)	Participants, n (% female); age (years; mean, SD)	Details	Group size	Weeks (contact hours)
Bogosian et al, 2015 [45]	Assess web-based MBCT[a] to reduce distress related to multiple sclerosis	• 21 (47.4); 53.4 (8.3)	• Web-based MBCT	• 5	• 8 (8)	19 (61.9); 50.9 (9.9)	WLC[b]	5	8 (8)
Cavalera et al, 2019 [46]	Assess web-based MBSR[c] to improve QOL[d], well-being, sleep, and fatigue for people with multiple sclerosis	• 54 (67); 42.3 (8.4)	• Web-based MBSR	• 6 (5 patients and 1 caregiver)	• 8 (16)	67 (62); 43.2 (9)	Web-based PED[e]	N/A[f]	8 (NI[g])
Hall et al, 2017 [47]	Compare phone vs live delivery of CBSM[h] among people with chronic fatigue syndrome	• 56 (92.9); 50.7 (11.1)	• Telephone CBSM	• NI	• 10 (15-20)	44 (86.4); 47.2 (8.3)	Live CBSM	NI	12 (18-24)
Heckman et al, 2006 [48]	Assess a telephone-delivered coping improvement group intervention on improving QOL in adults living with HIV or AIDS	• 44 (36.4); 53.5 (3.9)	• Telephone-delivered coping group	• 6-8	• 12 (18)	46 (28.3); 54.7 (3.7)	WLC	6-8	12 (18)

Heckman and Carlson, 2007 [49]	Assess 2 telephone-delivered mental health interventions for persons living with HIV or AIDS in rural United States	• 84 (25); 43.7 (8.3)<br>• 108 (35.2); 43.7 (9.1)	• Information support<br>• Coping improvement	• 8<br>• 8	• 8 (12)<br>• 8 (12)	107 (28); 41.9 (8.2)	UC[i] (all services provided by AIDS service organization, eg, case management and support groups, as well as assistance obtaining housing, employment, and childcare)	N/A	N/A
Heckman et al, 2013 [50]	Compare 2 telephone-delivered group therapies in reducing depressive symptoms among older adults infected with HIV	• 118 (37); 58.8 (5.4)<br>• 122 (48); 59.5 (4.9)	• Coping effective-ness<br>• Supportive-expressive	• 6-8<br>• 6-8	• 12 (18)<br>• 12 (18)	121 (31); 58.7 (5.0)	UC (community-based support services such as AIDS-related support groups, *12-step* programs, and individual therapy)	N/A	N/A
Hum et al, 2019 [51]	Examine outcomes of a distance-delivery group intervention program designed to improve emotional well-being in people with epilepsy	• 20 (80); 36.9 (2.9)<br>• 24 (62.5); 37.2 (2.6)	• UPLIFT[j]<br>• EpINFO[k]	• 6-8<br>• 6-8	• 8 (8)<br>• 8 (8)	15 (72.7); 29.4 (2.3)	WLC	6-8	8 (8)
Lepore et al, 2014 [52]	Compare the mental health benefits of supporting others using an enhanced prosocial ISG[l] vs a standard ISG	• 88 (100); 51.8 (8.4)	• Enhanced Prosocial ISG	• 14-17	• 6 (9)	95 (100); 52.7 (6.9)	Standard ISG	14-17	6 (9)

Marziali and Donahue, 2006 [53]	Assess an internet-based psychosocial intervention for family caregivers	• NI (76); NI	• Caring for others	• 4-6	• 10 (10) and 12-week peer support	NI (76); NI	No intervention	N/A	N/A
Park et al, 2020 [54]	Assess web-based 3RP[m] for parents of children with learning and attentional disabilities	• 31 (83.9); 47 (6.2)	• Web-based 3RP	• NI	• 8 (12) and intake	23 (100); 47 (4.9)	WLC	NI	8 (12) and intake
Paxton et al, 2007 [55]	Compare the outcomes of FTF[n] vs internet delivery of an intervention for body dissatisfaction and disordered eating in adult women	• 37 (100); 24.6 (SD 0.57)[o] • 42 (100); 27.2 (0.67)[o]	• Internet • FTF	• NI • NI	• 8 (12) • 8 (12)	37 (100); 24.8 (0.62)[o]	WLC	NI	8 (12)
Thompson et al, 2010 [56]	Evaluate a distance-delivered depression intervention for people with epilepsy	• 26 (77); 36.4 (34)	• Telephone intervention • Internet intervention	• 13 • 12	• 8 (8) • Self-guided	27 (85); 35.4 (31)	1. WLC: telephone; 2. WLC: internet	1. 13; 2. 12	1. 8 (8); 2. Self-guided
Thompson et al, 2015 [57]	Evaluate an MBCT intervention for preventing major depressive disorder episodes in people with epilepsy	• 64 (65.3); 41.2 (8.17)[o]	• UPLIFT	• ≥7	• 8 (8)	64 (65.3); 41.2 (8.17)[o]	UC (antidepressant medication and psychotherapy)	N/A	N/A
Van der Zanden et al, 2012 [58]	Evaluate a guided web-based CBT[p] course for young people with	• 121 (83.5); 20.8 (2.2)	• Web-based CBT	• ≥6	• 6 (9)	123 (85.4); 21 (2.3)	WLC	N/A	N/A

	depressive symptoms								
Vazquez et al, 2017 [59]	Assess CBT through conference call for nonprofessional caregivers	• 20 (90); 59.3 (9.7)<br>• 2. 22 (100); 59.7 (8.1)	• CBT<br>• Behavioral activation	• 5<br>• 5	• 5 (7.5)<br>• 5 (7.5)	19 (89.5); 55.9 (5.4)	UC (medical or psychological treatment in community)	N/A	N/A
Vranceanu et al, 2016 [60]; Zale et al, 2018 [65]	Assess web-based 3RP for neurofibromatosis vs PED control	• 32 (71); 42.9 (13.5)	• Web-based 3RP	• 6-8	• 8 (12)	31 (75); 39.9 (11.2)	Web-based PED	6-7	8 (12)
Wakefield et al, 2016 [61]	Assess web-based CBT intervention to assist parents of children who have completed cancer treatment	• 23 (91.3); 42 (5.8)	• Web-based CBT	• 3-5	• 3 (6)	22 (81.8); 42.8 (5.2)	WLC	3-5	3 (6)
Winter and Gitlin, 2007 [62]	Evaluate telephone-based support groups for female family caregivers of community-dwelling patients with dementia	• 58 (100); 68.7 (9.3)	• Telephone support group	• 5	• 26 (26)	45 (100); 64 (8.2)	UC (letter describing group assignment and next study steps)	N/A	N/A
Zernicke et al, 2014 [64]	Assess a web-based synchronous MBCR[q] group program for cancer survivors	• 30 (73.3); 58 (8.2)	• Web-based MBCR	• 4-11	• 8 (22)	32 (71.9); 58 (13)	WLC	4-11	N/A
Zerwas et al, 2016 [63]	Compare webchat-based CBT group therapy for bulimia vs traditional FTF group therapy	• 89 (98); 28.5 (9.3)	• Web-based CBT	• 3-5	• 20 (30)	90 (98); 27.5 (9.1)	FTF CBT	3-5	20 (30)

[a]MBCT: mindfulness-based cognitive therapy.
[b]WLC: wait list control.
[c]MBSR: mindfulness-based stress reduction.
[d]QOL: quality of life.
[e]PED: psychoeducation.
[f]N/A: not applicable.
[g]NI: no information.
[h]CBSM: cognitive behavioral stress management.
[i]UC: usual care.
[j]UPLIFT: using practice and learning to increase favorable thoughts.
[k]EpINFO: epilepsy information.
[l]ISG: internet support group.
[m]3RP: relaxation response resiliency program.
[n]FTF: face to face.
[o]SD has been calculated as it was not provided in the cited study.
[p]CBT: cognitive behavioral therapy.
[q]MBCR: mindfulness-based cancer recovery.
