# Supplementary material for: Effectiveness of Live Health Professional–Led Group eHealth Interventions for Adult Mental Health: Systematic Review of Randomized Controlled Trials
Source: J Med Internet Res. 2022 Jan 11;24(1):e27939. doi: 10.2196/27939 (PMC8790691; doi:10.2196/27939)
Supplement: Multimedia Appendix 4 [file jmir_v24i1e27939_app4.pdf]

Appendix 4. Risk of bias screening results

Study	Domain 1	Domain 2	Domain 3	Domain 4	Domain 5	Overall
Bogosian et al. (2015)	Low	Some Concerns[d,f]	Low	High[l]	Some Concerns[n]	High
Cavalera et al. (2019)	Some Concerns[b]	High[d,e,f]	High[g,i]	Some Concerns[k]	High[m]	High
Hall et al. (2017)	Some Concerns[a,b]	Some Concerns[d,f]	Low	Some Concerns[k]	Some Concerns[n]	Some Concerns
Heckman et al. (2006)	Some Concerns[b]	Low	Some Concerns[j]	High[l]	Some Concerns[n]	High
Heckman et al. (2007)	Some Concerns[a,b]	Low	Low	Some Concerns[k]	Some Concerns[n]	Some Concerns
Heckman et al. (2013)	Some Concerns[b]	Low	High[h,i,g]	Some Concerns[k]	Some Concerns[n]	High
Hum et al. (2019)	Some Concerns[b]	Some Concerns[d,f]	High[g,i]	High[l]	Some Concerns[n]	High
Lepore et al. (2014)	Low	Low	Low	Some Concerns[k]	Some Concerns[n]	High
Marziali et al. (2006)	Some Concerns[a,b]	High[d,e]	High[g,h]	High[l]	Some Concerns[n]	High
Park et al. (2020)	High[b,c]	High[d,e,f]	High[g,i]	High[l]	Some Concerns[n]	High
Paxton et al. (2007)	Some Concerns[b]	Low	High[g,i]	High[l]	Some Concerns[n]	High
Thompson et al. (2010)	Some Concerns[a,b]	High[d,e,f]	High[g,h,i]	High[l]	Some Concerns[n]	High
Thompson et al. (2015)	Some Concerns[a,b,c]	Some Concerns[d,f]	High[g,h,i]	High[l]	Some Concerns[m]	High
Van der Zanden et al. (2012)	Some Concerns[b]	Some Concerns[d,f]	Low	High[l]	Some Concerns[n]	High
Vazquez et al. (2017)	Low	Some Concerns[d,f]	Some Concerns[g]	High[l]	Some Concerns[m]	High
Vranceanu	Some	Some	Low	Some	Some	Some

et al. (2016)	Concerns[b]	Concerns[f]		Concerns[k]	Concerns[n]	Concerns
Wakefield et al. (2016)	Low	High[d,e]	High[g,h,i]	High[l]	High[m]	High
Winter et al. (2007)	Some Concerns[a,b]	Some Concerns[d,e,f]	Some Concerns[j]	High[l]	Some Concerns[n]	High
Zale et al. (2018)	Some Concerns[b]	Some Concerns[f]	High[h,i]	Some Concerns[k]	Some Concerns[n]	High
Zernicke et al. (2014)	Low	Some Concerns[d,f]	Low	High[l]	Low	High
Zerwas et al. (2016)	Low	Some Concerns[d,f]	High[g,h]	High[l]	Some Concerns[n]	High
